# Selected toxic and essential heavy metals in impacted teeth and the surrounding mandibular bones of people exposed to heavy metals in the environment

**DOI:** 10.1186/s12995-016-0146-1

**Published:** 2016-12-12

**Authors:** Piotr Malara, Agnieszka Fischer, Beata Malara

**Affiliations:** 1 Institute of Engineering Materials and Biomaterials, Silesian University of Technology, 18a Konarskiego str, 44-100 Gliwice, Poland; 2 Department of Toxicology, Medical University of Silesia, 4 Jagiellonska str, 41-200 Sosnowiec, Poland; 3 Institute of Medicine, Katowice School of Economics, 3 Harcerzy Wrzesnia str, 40-659 Katowice, Poland

**Keywords:** Tooth chemistry, Bone, Heavy metals, Cadmium, Lead, Atomic absorption spectrometry, Biomonitoring, Environmental exposure

## Abstract

**Background:**

The elemental composition of bones and teeth can allow exposure to heavy metals in the environment to be estimated. The aim of this study was to determine whether impacted mandibular teeth and the surrounding bones can be used as biomonitoring media to assess exposure to heavy metals.

**Methods:**

The research materials were 67 impacted lower third molars and samples of the cortical bone removed when the wisdom teeth were surgically extracted. The samples were from people living in two areas with different environmental concentrations of heavy metals. The cadmium, chromium, copper, iron, lead, manganese, and zinc concentrations in the samples were determined by atomic absorption spectrometry with flame atomization.

**Results:**

The cadmium and lead concentrations in the impacted third molars and the bones surrounding the teeth were significantly higher for people living in the relatively polluted Ruda Slaska region than for people living in Bielsko-Biala region. Significantly higher chromium, copper, manganese, and zinc concentrations were found in the bones surrounding the impacted teeth from people living in Ruda Slaska than in the bones surrounding the impacted teeth from people living in Bielsko-Biala. The cadmium concentrations in impacted teeth and the surrounding bones were significantly positively correlated.

**Conclusion:**

The results indicated that impacted mandibular teeth and the surrounding mandibular bones may reflect the exposure of people to cadmium and lead in the environment. This conclusion, however, must be verified in future research projects designed to exclude the possibility of additional dietary, occupational, and other types of exposure to heavy metals.

## Background

The elemental compositions of the bones and teeth of a person can provide information on the diet and health of that person and the exposure of the person to chemicals in the environment [1–3]. In the field of forensic medicine, research has been performed on the use of the elemental compositions of bones and teeth to identify murder victims and ancient human remains [4]. Links between the concentrations of certain metallic elements in children’s teeth and behavioural deficits have been described [5].

Bones and teeth are calcified tissues, and hydroxyapatite is the main component of both. Bones and teeth are chemically stable, although it is believed that certain trace elements may accumulate in them by replacing calcium in the hydroxyapatite structure [6].

Deciduous teeth are mainly used in environmental studies because they are easily obtained after being naturally shed. Information on exposure to metals in the environment during childhood can be provided by deciduous teeth [7, 8]. Deciduous teeth start to develop *in utero*, and the last deciduous teeth are replaced with permanent teeth when a child is 12–13 years of age [9]. Deciduous and permanent teeth are morphologically and histologically similar, so environmental exposure to metals may also be assessed by analysing permanent teeth [10]. However, the availability of permanent teeth for analysis is a problem because it is unacceptable to extract permanent teeth for biomonitoring purposes. It should be noted that tooth enamel is constantly in direct contact with the environment of the oral cavity. This means that many physiological and pathological factors that affect the oral cavity (such as the carious process, the consumption of food, and the use of drugs) can affect the elemental compositions of the surface layers of teeth [11].

Although hydroxyapatite is the main component of teeth and bones, the structures of teeth and bones have different histological remodelling dynamics [12]. At the structural level, there are two types of bone, cortical and cancellous bone. Cancellous bone can provide information on recent exposure to metals, whereas cortical bone can accumulate trace elements over much longer periods [13]. Determining the elemental composition of cortical bone is preferred to determining the elemental composition of cancellous bone because cortical bone has a compact structure and is less sensitive to contamination during sampling and sample preparation [14]. Generally speaking, concentrations of trace elements are usually higher in cancellous bone than in cortical bone. Similarly, concentrations of trace elements are usually higher in the metaphyseal areas than in the shafts of long bones [15].

No indications have been reported of elements in the mineralogical structures of a tooth being released once the tooth has been formed [12]. In contrast, elements can be released from bone into the blood in a range of physiological and pathological situations [13].

### Knowledge gaps

An impacted tooth is a fully developed tooth that has, for any of a number of reasons, not erupted and has remained entirely covered in bone tissue in the jaw [16]. Impacted teeth could be valuable for use in biomonitoring studies. An impacted tooth is isolated from the oral cavity, and blood is the only source of elements to the structure of such a tooth. The third molars in the mandible are the most commonly impacted teeth. The third molars in the mandible become completely developed when a person reaches an age of between 17 and 21 years, when all of the other teeth are present in the dental arch and bone growth is complete [9]. It is very often necessary to surgically remove impacted teeth for orthodontic or surgical reasons [17]. Removed impacted teeth are waste materials that can be used in research studies. The elemental compositions of impacted teeth are of particular interest for the reasons given above. A certain amount of overlying bone has to be removed to allow an impacted tooth to be extracted [18]. It is possible, with skilful surgery, to acquire both impacted teeth and overlying bone samples suitable for analysis. This allows calcified tissues from the same anatomical area but with different histological and physiological characteristics to be analysed. An impacted tooth and the overlying bone structure will in fact have been in direct contact with each other over the lifetime of the individual to whom they belong.

The mandibular bone has an unusual anatomy, with a relatively thick layer of cortical bone and very little cancellous bone. An impacted tooth will be covered almost exclusively with cortical bone [16, 17]. The overlying bone may be collected when an impacted tooth is removed, and the elemental compositions of the bone and tooth can be determined [18].

No information is currently available on whether heavy metal concentrations in an impacted tooth and the surrounding mandibular bone reflect heavy metal concentrations in the environment. It is difficult to acquire such information because heavy metals in tooth and bone structures can come from a range of different types of exposure that can occur simultaneously.

Potential interactions between elements within the structures of calcified tissues mean that it is necessary to determine both toxic and essential elements when biomonitoring studies are performed to investigate the effects of environmental or occupational exposure on the accumulation of elements in tissues [19].

### Aim of the study

The aim of the study was to determine whether impacted mandibular teeth and the surrounding bones reflect environmental exposure to heavy metals and can be used as biomonitoring materials. To achieve this, cadmium, chromium, copper, iron, lead, manganese, and zinc were determined in samples of impacted teeth and the surrounding bone from people who will have been exposed to different ranges and concentrations of heavy metals where they live.

## Methods

### Patients and samples

**Fig. 1:**
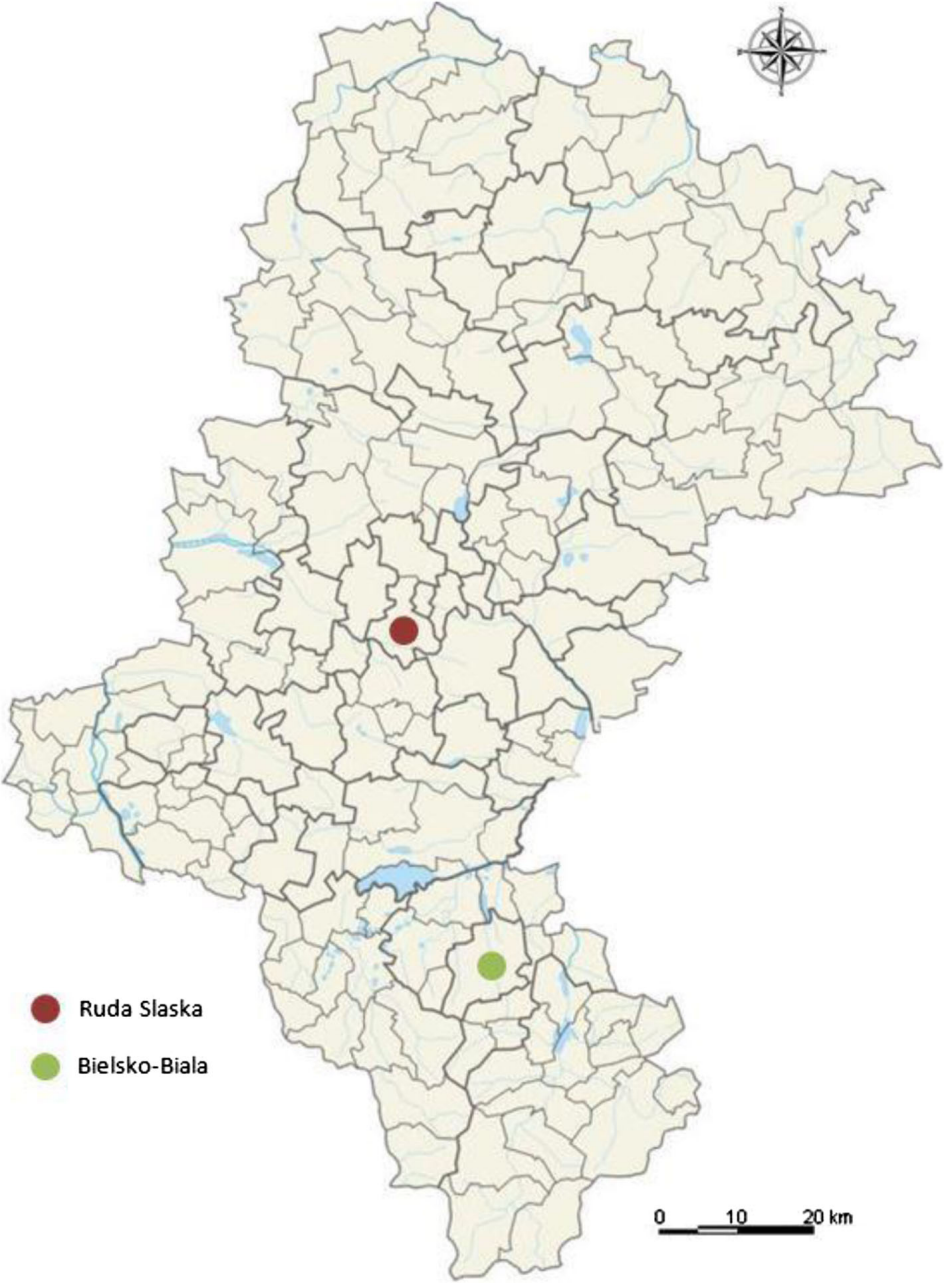
A section of a map of Poland showing the location of Ruda Slaska and Bielsko-Biala

The material used in the study consisted of 67 impacted lower third molars and 67 samples of the mandibular cortical bones surrounding the impacted teeth. The samples came from people from 18 to 40 years of age. Each person supplied one tooth and one bone sample. The samples were collected from 23 women and 11 men living in Ruda Slaska and 23 women and 10 men living in Bielsko-Biala. Ruda Slaska and Bielsko-Biala are cities in southern Poland, 52 km apart, and polluted with heavy metals to different degrees (see Table 1) [20, 21]. A map showing the locations of Ruda Slaska and Bielsko-Biala is shown in Fig. 1.

**Table 1:** The average annual PM 10, Cd in PM 10 and Pb concentrations in the air in Bielsko-Biala and Ruda Slaska [20]

	Year	2015^a^	2014^a^	2006	2005	2004	2003	2002
Pb [μg/m^3^]	Bielsko-Biala	0,024 ± 0,021	0,027 ± 0,021	0,04	0,02	0,04	0,05	0,04
	Ruda Slaska	0,040 ± 0,015^b^	0,044 ± 0,022^b^	0,07	0,06	0,05	0,08	0,07
Cd in PM10 [ng/m^3^]	Bielsko-Biala	0,58 ± 0,41	0,65 ± 0,53	N/A	N/A	N/A	N/A	N/A
	Ruda Slaska	0,84 ± 0,45^b^	1,40 ± 1,21^b^	N/A	N/A	N/A	N/A	N/A
PM10 [μg/m^3^]	Bielsko-Biala	36,80 ± 14,54	37,92 ± 14,61	0-36	0-43	40-58	35-43	30-42
	Ruda Slaska	39,17 ± 14,30^b^	43,33 ± 15,36^b^	40-60	46-56	31-57	41-72	41-63

^a^data obtained from the database of Regional Inspectorate for Environmental Protection in Katowice (Wojewodzki Inspektorat Ochrony Srodowiska in Katowice) http://powietrze.katowice.wios.gov.pl

^b^data from the air quality monitoring station in Katowice

Ruda Slaska has a population of more than 140,000, and it is in the “Upper Silesian industrial district” in which high heavy metals concentration have repeatedly been found in the air for many years [20]. The air quality is considered to be one of the best factors representing the overall environmental burden and having the most important impact on human health [22]. The economy of Ruda Slaska has been profoundly restructured, but heavy industry is still a crucial part of the economy. Bielsko-Biala has a population of more than 170,000, and is the capital of Podbeskidzie. Bielsko-Biala has a strong tourism industry and a relatively clean environment. Pollutants in Bielsko-Biala mainly come from local “low emission” sources and emissions in Slovakia and the Czech Republic transported in the atmosphere. Although, the region of Bielsko-Biala is known to have raising environmental issues concerning mainly the air quality, the data shown in Table 1 indicate that lead and cadmium in PM10 levels in the air of Bielsko-Biala are lower than concentrations of these heavy metals in the air of Ruda Slaska (0,024 ± 0,021 vs. 0,040 ± 0,015 for Pb and 0,58 ± 0,41 vs. 0,84 ± 0,45 for Cd in PM10 in year 2015; and 0,027 ± 0,021 vs. 0,044 ± 0,022 for Pb and 0,65 ± 0,53 vs. 1,40 ± 1,21 for Cd in PM10 in year 2014). It must be remembered that human teeth represent a long term accumulation of heavy metals. As the mean age of the population under the study (25,7 ± 7,2 y) and the periods of time when the wisdom teeth are formed and the rate of mineralization is the highest (between 12 and 17 years of age), Table 1 contains the historical data showing the lead levels in the air of Bielsko-Biala and Ruda Slaska in years 2002–2006. In those years the lead levels in the air of Upper Silesia Region were higher than the lead levels in the air of Bielsko-Biala. Taking into account the data shown in Table 1, we decided to use Bielsko-Biala as a reference area. As we intended to limit to the lowest possible level the influence of industry and motor traffic, only samples from people who lived from 6 to 14 km from the city centre were analysed. It must be emphasised that the area was not free from long-range emission, but free of industry with typical occupational setting in agriculture and paperwork. The participants from Bielsko-Biala were grouped into those who lived 6–10 km from the city centre (21 people) and those who lived 10–14 km from the city centre (12 people).

Each participant in the study declared that he or she had lived in either Ruda Slaska or Bielsko-Biala since birth and had never left that area for more than 2 months. All of the participants declared that they were non-smokers and had not been exposed to cigarette smoke at home (i.e., they were not passive smokers). None of the participants regularly took medication or dietary supplements. None of the participants had ever worked in an industry known to increase exposure to heavy metals. All of the participants confirmed that they were not on special diets, that they bought their food from shops, and that they did not use any food grown on their own land. The medical histories of the participants were taken into consideration, but no additional medical examinations were performed. The participants were asked about their education levels, and the participants were divided into four groups: those who had only primary education, those who had vocational education, those who had secondary education, and those who had higher education. The research project was approved by the Silesian Medical University ethics committee.

The biological samples were collected when the participants had their impacted lower third molars surgically removed at an oral surgery clinic. Each procedure was carried out under local anaesthetic by a specialist oral and maxillofacial surgeon. The mandibular bone covering an impacted tooth was cut out using a piezo-surgical unit, then the tooth was extracted using elevators and forceps. Immediately after removal, the tooth and bone samples were washed with double-distilled water, then each sample was placed in a tightly sealed Teflon vessel and stored until it was prepared for analysis.

The samples were prepared following a chromium analysis procedure that has been described previously [23]. Each sample was dried at 105 °C until a constant weight was reached. Each sample was then pulverized, and the powder produced was stored in a desiccator. Each powdered sample was dried again at 105 °C for 4 h before being dissolved.

An aliquot of between 0.500 and 1.000 g of a dried and pulverized bone or tooth sample was dissolved in 3 mL of ultrapure concentrated nitric acid (Supra-Merck 65%; Merck, Darmstadt, Germany) in a Teflon vessel for 3 h at 120 °C. The clear solution produced was diluted to 10 mL with double-distilled water. All of the chemicals that were used were of the highest purity available.

### Heavy metal analysis

The cadmium, chromium, copper, iron, lead, manganese, and zinc concentrations in the sample solutions were determined by flame atomization atomic absorption spectrometry using a Philips PYE Unicam SP-9 instrument (Philips, Amsterdam, The Netherlands). An air–acetylene mixture was used, and the absorption lines used for cadmium, chromium, copper, iron, lead, manganese, and zinc were 228.8, 357.9, 324.8, 248.3, 283.3, 279.5, and 231.9 nm, respectively.

Teeth and bones are histologically similar, so a certified bone meal reference material (SRM 1486; US National Institute of Standards and Technology, Gaithersburg, MD, USA) was analysed for cadmium, copper, iron, lead, manganese, and zinc and a certified bone ash reference material (SRM 1400; US National Institute of Standards and Technology) was analysed for chromium. The analyte concentrations we found and the certified concentrations are shown in Table 2. The precision of the method was close to 8%, and the accuracies for the different elements were between 3.01% and 6.99%.

**Table 2:** Certified and found heavy metal concentrations (μg/g) in the reference materials

Element	Certified value	Experimental value
Pb	1.335 ± 0.014	1.483 ± 0.024
Cd	0.011^a^	0.013 ± 0.010
Fe	99 ± 8	112 ± 14
Mn	1.16^a^	1.32 ± 0.36
Cr	0.03^b^	0.03 ± 0.010
Cu	0.8^a^	0.91 ± 0.07
Zn	147 ± 16	141 ± 12

SRM 1486 bone meal (Cd, Cu, Fe, Mn, Pb, and Zn concentrations) and SRM 1400 bone ash (Cr concentrations); both reference materials were supplied by the US National Institute of Standards and Technology (Gaithersburg, MD, USA)

^a^non-certified value

^b^SRM 1400 bone ash; non-certified value

### Statistical analysis

The data for the teeth and bone samples were grouped by the area the donors of the samples lived in before statistical analyses were performed. The Kolmogorov–Smirnov goodness-of-fit test and plots of the results showed that concentrations of all the metals were not normally distributed. Subjecting the data to a natural logarithmic transformation caused the distributions to become normal. This allowed parametric tests to be used. The test procedures that were used have been described previously [24]. Student’s *t*-test was performed to identify significant differences between the mean metal concentrations in the samples provided by people living in Bielsko-Biala and Ruda Slaska. Data have been treated in the same way in previous studies [8, 25, 26]. The ratios between the metal concentrations in impacted teeth and the surrounding bones (Me_(tooth)_/Me_(bone)_) were calculated, and Student’s *t*-test was performed to identify significant differences between the ratios for the samples provided by people living in Bielsko-Biala and Ruda Slaska. Pearson’s product–moment test was used to identify correlations between each element in the tooth and bone samples. Different numbers of samples were provided by men and women, so the non-parametric Mann–Whitney *U* test was used to determine the significances of the differences between the metal concentrations in the samples provided by men and women. Relatively few samples were analysed, so non-parametric tests were also used to determine the significances of the differences between the metal concentration in the samples provided by people with different educational backgrounds (for which the Kruskall–Wallis test was used) and between the metal concentrations in the samples provided by people living different distances from the Bielsko-Biala city centre (for which the Mann–Whitney *U* test was used). The statistical analyses were performed using Statistica 10.0 software (Statsoft, Tulsa, OK, USA). The statistical tests were performed using the 95% level to indicate a significant result.

## Results

The 67 participants were between 18 and 40 years old. The mean ages (± the standard deviation) of the participants from Ruda Slaska and Bielsko-Biala were 24.9 ± 5.9 y (25.3 ± 5.7 years for women and 24.7 ± 6.2 years for men) and 26.1 ± 8.7 y (26.8 ± 7.9 years for women and 25.4 ± 8.5 years for men), respectively.

The mean concentrations of the seven metals that were analysed in the teeth and the bone surrounding the teeth provided by the people from the two different study areas are shown in Table 3. The Student’s *t*-test results (the *p*-levels are shown in Tables 3) indicated that the cadmium and lead concentrations were significantly higher in the tooth and bone samples provided by people living in Ruda Slaska than in the samples provided by people living in Bielsko-Biala. The chromium, copper, manganese, and zinc concentrations were also significantly higher in the bone samples provided by the Ruda Slaska citizens than in the bone samples provided by the Bielsko-Biala citizens. The chromium and manganese concentrations in the bone samples from men from Ruda Slaska were significantly different from the chromium and manganese concentrations provided by women from Ruda Slaska (Table 4). There were no statistically significant differences between the metal concentrations in the impacted teeth from men and women living in Ruda Slaska (Table 4) and between the metal concentrations in bone and tooth samples from men and women living in Bielsko-Biala (Table 5). However, the reason(s) for these differences could not be identified because of the small numbers of samples provided.

**Table 3:** Descriptive statistics for the heavy metal concentrations (μg/g) in impacted wisdom teeth and in the bone surrounding the impacted teeth

		Samples from people living in Bielsko-Biala (*n* = 33)			Samples from people living in Ruda Slaska (*n* = 34)			*p* - level^*^
	Sample	Mean ± standard deviation	Min - Max	95% conf. limits of mean	Mean ± standard deviation	Min - Max	95% conf. limits of mean	
Pb	Teeth	10.29 ± 1.90	6.24 - 14.26	9.09 - 12.49	13.23 ± 1.93	6.26 - 18.10	11.21-15.25	0.038^a^
	Bone	3.53 ± 2.02	1.04 - 8.44	2.85 - 4.28	7.95 ± 2.66	2.06 - 13.30	7.02 - 8.88	<0.001^a^
Cd	Teeth	0.10 ± 0.02	0.07 - 0.14	0.09 - 0.12	0.12 ± 0.02	0.08 - 0.15	0.12 - 0.13	0.006^a^
	Bone	0.68 ± 0.36	0.05 - 1.38	0.55 - 0.81	0.97 ± 0.42	0.10 - 1.84	0.82 - 1.11	0.004^a^
Fe	Teeth	10.31 ± 1.99	7.14 - 14.87	9.60 - 11.01	10.48 ± 1.85	7.80 - 14.24	9.83 - 11.12	0.677
	Bone	19.08 ± 12.69	4.24 - 48.16	14.58 - 23. 58	17.61 ± 9.23	3.74 - 45.57	14.39 - 20.83	0.588
Mn	Teeth	3.58 ± 0.45	2.99 - 4.52	3.42 - 3.74	3.76 ± 0.45	2.94 - 4.69	3.60 - 3.92	0.960
	Bone	1.64 ± 1.10	0.12 - 4.03	1.25 - 2.03	4.05 ± 2.36	0.29 - 7.17	3.23 - 4.87	<0.001^a^
Cr	Teeth	5.73 ± 1.65	3.06 - 8.36	5.14 - 6.32	5.54 ± 1.57	3.63 - 8.67	4.99 - 6.09	0.767
	Bone	1.76 ± 1.70	0.19 - 13.74	0.96 - 2.56	3.79 ± 4.20	0.15 - 14.72	2.33- 5.26	0.016^a^
Cu	Teeth	8.83 ± 1.30	6.02 - 11.58	8.37 - 9.29	9.13 ± 1.11	6.02 -11.30	8.75 - 9.52	0.359
	Bone	6.86 ± 3.87	1.49 - 15.60	5.49 - 8.23	13.77 ± 7.73	4.39 - 27.74	11.07 - 16.47	<0.001^a^
Zn	Teeth	297.31 ± 44.73	201.36 - 382.02	281.44 - 313.17	314.87 ± 59.37	208.54 - 439.30	294.16 - 335.59	0.113
	Bone	155.76 ± 38.38	72.99 - 237.02	142.15 - 169.36	229.10 ± 107.96	85.78 - 443.74	191.43 - 266.77	<0.001^a^

*n* number of samples

^*^ *p-*levels, determined using Student’s *t*-tests, used to identify statistically significant differences between the mean heavy metal concentrations in the samples provided by people living in Bielsko-Biala and Ruda Slaska

^a^ indicates the heavy metal concentrations in the samples provided by people living in Bielsko-Biala and Ruda Slaska were significantly different

**Table 4:** Heavy metal concentrations (μg/g) in the samples provided by women and men living in Ruda Slaska

	Impacted teeth (*n* = 34)				Bone surrounding the impacted teeth (*n* = 34)			
Element	Women (*n* = 23) Median	Men (*n* = 11) Median	U-value	*p* - level^*^	Women (*n* = 23) Median	Men (*n* = 11) Median	U-value	*p* - level^*^
Pb	9.30	8.83	118	0.768	8.05	6.63	91	0.197
Cd	0.12	0.13	99	0.320	0.86	0.87	111	0.580
Fe	9.99	9.99	120	0.839	15.32	20.84	92	0.210
Mn	3.85	3.84	125	0.970	5.29	1.70	66	0.027^a^
Cr	4.72	5.79	83	0.113	4.47	1.31	51	0.006^a^
Cu	9.02	9.10	114	0.658	15.30	8.08	68	0.052
Zn	318.15	284.26	107	0.484	221.19	160.61	67	0.059

*n* number of samples

^*^ *p*-levels, estimated using the Mann–Whitney U test, used to identify statistically significant differences between the metal concentrations in the samples provided by women and men

^a^ indicates the mean concentrations in the samples provided by women and men were significantly different

**Table 5:** Heavy metal concentrations (μg/g) in the samples provided by women and men living in Bielsko-Biala

	Impacted teeth (*n* = 33)				Bone surrounding the impacted teeth (*n* = 33)			
Element	Women (*n* = 23) Median	Men (*n* = 10) Median	U-value	*p* - level^*^	Women (*n* = 23) Median	Men (*n* = 10) Median	U-value	*p* - level^*^
Pb	10.03	11.54	95.50	0.456	4.04	1.78	95.00	0.444
Cd	0.11	0.12	87.50	0.290	0.71	0.50	85.00	0.247
Fe	10.52	8.87	67.50	0.065	14.18	18.92	102.00	0.624
Mn	3.65	3.26	96.50	0.480	1.57	0.99	92.00	0.378
Cr	4.72	6.84	59.50	0.061	1.45	0.99	86.00	0.264
Cu	8.86	8.72	106.50	0.753	7.36	3.98	88.00	0.299
Zn	282.67	306.77	86.50	0.272	162.60	160.36	104.00	0.680

*n* the number of samples

^*^ *p*-levels, estimated using the Mann–Whitney U test, used to identify statistically significant differences between the metal concentrations in the samples provided by women and men

No statistically significant differences were found in the metal concentrations in the teeth or bones from people living different distances from the Bielsko-Biala city centre (Table 6). No significant differences were found between the metal concentrations in samples from people with different educational levels in either Ruda Slaska or Bielsko-Biala (Tables 7 and 8).

**Table 6:** Heavy metal concentrations (μg/g) in the samples from people living different distances from the centre of Bielsko-Biala

	Impacted teeth (*n* = 33)				Bone surrounding the impacted teeth (*n* = 33)			
Element	6-10 km (*n* = 21) Median	10-14 km (*n* = 12) Median	U-value	*p* - level^*^	6-10 km (*n* = 21) Median	10-14 km (*n* = 12) Median	U-value	*p* - level^*^
Pb	10.79	10.03	125.50	0.125	3.74	4.36	88	0.160
Cd	0.10	0.11	87.00	0.149	0.62	0.77	85	0.129
Fe	11.32	8.98	81.50	0.099	14.52	13.53	101	0.359
Mn	3.45	3.42	104.00	0.421	1.14	1.69	98	0.312
Cr	4.68	5.39	100.50	0.349	1.29	1.49	104	0.421
Cu	8.79	8.86	113.50	0.653	7.09	5.91	125	0.985
Zn	293.94	294.23	98.50	0.312	170.71	156.67	125	0.985

*n* number of samples

^*^ *p*-levels, estimated using the Mann–Whitney U test, used to identify statistically significant differences between the metal concentrations in the samples provided by people living different distances from the centre of Bielsko-Biala

**Table 7:** Heavy metal concentrations (μg/g) in the samples from people with different levels of education living in Ruda Slaska

	Impacted teeth (*n* = 34)						Bone surrounding the impacted teeth (*n* = 34)					
	Educational level						Educational level					
Element	Primary (*n* = 6) Median	Vocational (*n* = 8) Median	Secondary (*n* = 11) Median	Higher (*n* = 9) Median	H-value	*p* - level^*^	Primary (*n* = 6) Median	Vocational (*n* = 8) Median	Secondary (*n* = 11) Median	Higher (*n* = 9) Median	H-value	*p* - level^*^
Pb	14.63	12.41	11.89	11.83	9.88	0.196	7.69	7.22	7.80	8.04	1.171	0.982
Cd	0.12	0.12	0.14	0.11	7.90	0.481	0.64	0.86	1.20	0.91	4.112	0.249
Fe	9.14	9.74	9.94	11.92	7.47	0.098	25.45	18.69	15.32	13.37	7.772	0.873
Mn	3.84	3.84	3.85	3.94	1.58	0.901	3.82	3.42	4.23	3.91	1.653	0.884
Cr	5.09	5.79	4.66	5.79	4.32	0.229	1.34	1.99	4.47	1.45	4.121	0.249
Cu	8.60	9.05	9.84	8.77	1.58	0.664	10.40	10.56	15.08	8.45	1.980	0.577
Zn	356.55	274.80	288.92	321.86	4.65	0.207	136.11	187.74	212.39	210.62	2.691	0.442

*n* number of samples

^*^ *p*-levels, estimated using the ANOVA Kruskal–Wallis test, used to identify statistically significant differences between the metal concentrations in the samples provided by people with different levels of education living in Ruda Slaska

**Table 8:** Heavy metal concentrations (μg/g) in the samples from people with different levels of education living in Bielsko-Biala

	Impacted teeth (*n* = 33)						Bone surrounding the impacted teeth (*n* = 33)					
	Educational level						Educational level					
Element	Primary (*n* = 7) Median	Vocational (*n* = 11) Median	Secondary (*n* = 9) Median	Higher (*n* = 6) Median	H-value	*p* - level^*^	Primary (*n* = 7) Median	Vocational (*n* = 11) Median	Secondary (*n* = 9) Median	Higher (*n* = 6) Median	H-value	*p* - level^*^
Pb	14.08	13.20	12.02	11.70	5.126	0.162	3.44	3.81	4.54	4.11	3.460	0.326
Cd	0.12	0.11	0.12	0.10	2.109	0.549	0.34	0.65	0.84	0.67	13.959	0.096
Fe	8.72	9.92	8.79	11.94	7.660	0.536	13.44	13.16	12.05	16.70	8.352	0.046
Mn	3.26	3.89	3.19	3.73	6.063	0.109	0.86	1.22	1.59	1.52	2.054	0.561
Cr	5.95	5.39	4.72	4.54	2.956	0.398	0.86	1.45	1.96	1.12	4.016	0.259
Cu	8.25	8.86	8.86	9.16	2.208	0.530	6.93	7.09	8.52	4.76	2.025	0.567
Zn	328.10	263.94	282.67	293.94	6.303	0.097	101.80	172.83	162.60	165.19	11.124	0.091

*n* number of samples

^*^ *p*-levels, estimated using the ANOVA Kruskal–Wallis test, used to identify statistically significant differences between the metal concentrations in the samples provided by people with different levels of education living in Bielsko-Biala

Descriptive statistics for the Me_(tooth)_/Me_(bone)_ ratios are shown in Table 9. Student’s t-tests indicated that the differences between the ratios for the samples provided by people living in Ruda Slaska and Bielsko-Biala were not significantly different. Pearson’s correlation coefficients for the relationships between the metal concentrations in the tooth and bone samples are shown in Table 10. Only the cadmium concentrations in the tooth and bone samples positively correlated, and this relationship was only found for the samples provided by people living in Ruda Slaska.

**Table 9:** Descriptive statistics for the Me_(tooth)_/Me_(bone)_ ratios (unitless) for the samples provided by people living in Bielsko-Biala and Ruda Slaska

	Coefficients Me_(tooth)_/Me_(bone)_ for the biological material from people living in Bielsko-Biala (*n* = 33)			Coefficients Me_(tooth)_/Me_(bone)_ for the biological material from people living in Ruda Slaska (*n* = 34)			*p* - level^*^
	Mean ± standard deviation	Min - Max	95% conf. limits of mean	Mean ± standard deviation	Min - Max	95% conf. limits of mean	
Pb	4.89 ± 4.14	0.97 - 17.49	3.42 - 6.35	1.47 ± 0.78	0.65 - 4.29	1.19 - 1.74	0.254
Cd	0.32 ± 0.22	0.08 - 2.43	0.15 - 0.48	0.19 ± 0.11	0.07 - 1.43	0.10 - 0.27	0.081
Fe	0.82 ± 0.59	0.18 - 2.80	0.61 - 1.02	0.78 ± 0.52	0.20 - 2.95	0.60 - 0.97	0.438
Mn	3.93 ± 2.53	0.88 - 26.36	2.32 - 5.55	1.91 ± 1.40	0.44 - 11.80	1.07 - 2.75	0.078
Cr	6.04 ± 3.61	0.59 - 31.28	3.69 - 8.38	4.47 ± 3.14	0.31 - 33.52	2.32 - 6.62	0.295
Cu	1.98 ± 1.59	0.50 -7.01	1.41 - 2.54	0.92 ± 0.56	0.30 - 2.57	0.73 - 1.12	0.059
Zn	2.08 ± 0.81	1.12 - 4.50	1.80 - 2.37	1.68 ± 0.89	0.67 - 4.47	1.37 - 1.99	0.086

*n* number of samples

^*^ *p*-levels, estimated using Student’s *t*-test, used to identify statistically significant differences between the Me_(tooth)_/Me_(bone)_ ratios for the samples provided by people living in Bielsko-Biala and Ruda Slaska

**Table 10:** Pearson’s correlation coefficients describing relationships between metal concentrations in teeth and the bone surrounding the teeth

	Pb	Cd	Fe	Mn	Cr	Cu	Zn
Ruda Slaska	0.02	0.36^*^	-0.22	0.03	-0.04	-0.10	0.17
Bielsko-Biala	-0.14	-0.23	0.09	0.29	0.23	-0.05	-0.31

^*^ indicates the correlation was significant at *p* < 0.05

## Discussion

The exposure of an organism to heavy metals can be assessed by analysing a range of tissues. Most tissues, however, mainly provide information on recent exposure, and the metal concentrations can vary considerably depending on the time between exposure and sample collection. For environmental biomonitoring, it is important to analyse tissues that reflect chronic exposure. Heavy metal concentrations in bones and teeth (the main mineral component of which is hydroxyapatite) reflect chronic exposure. Mineral turnover will be much faster in bones than in teeth because of the morphological and physiological characteristics of the tissues, so bones will reflect more recent exposure to heavy metals than will teeth. It is hardly possible for metals to be released from the structures of teeth.

### Comparison with previous research

Teeth have been used to indicate environmental exposure to metals in many studies [27–29]. Deciduous teeth have mainly been used in such studies [7, 8]. Deciduous teeth meet the criterion that a good biomonitoring material should be easily accessible. In the study described here, we were able to use impacted permanent teeth and the surrounding mandibular bones as biomonitoring tissues.

We found differences in the cadmium and lead concentrations in the tooth samples provided by people living in the different study areas. The cadmium and lead concentrations in teeth provided by people living in Bielsko-Biala were 0.10 ± 0.02 and 10.29 ± 1.90 μg/g, respectively, and the cadmium and lead concentrations in teeth provided by people living in Ruda Slaska were 0.12 ± 0.02 and 13.23 ± 1.93 μg/g, respectively (Table 3). One reason for this could have been different environmental burdens of cadmium and lead in Bielsko-Biala and Ruda Slaska. Similar results have previously been found for deciduous teeth [7, 25] and permanent teeth [30–32]. In those studies, the cadmium and lead concentrations in teeth were found to reflect the cadmium and lead concentrations in different environmental compartments. The heavy metal concentrations in the samples of mandibular bone we analysed were within the concentration ranges found in other studies in which human bones have been analysed [4]. The mandibular bone samples we analysed reflected the cadmium, chromium, copper, lead, manganese, and zinc concentrations in environmental media in the areas in which the people who provided the samples lived.

A number of factors related to the sex of a person may affect heavy metal concentrations in the person’s teeth. These factors include hormone concentrations [33, 34] and tooth formation and eruption times [9]. However, no differences have been found in the concentrations of a number of elements in deciduous and permanent teeth in males and females in a number of studies [35–37]. We found no differences in the heavy metal concentrations in the impacted teeth from men and women from either Ruda Slaska or Bielsko-Biala (Tables 4 and 5, respectively). We found significant differences in the chromium and manganese concentrations in the mandibular bone samples from men and women living in Ruda Slaska (the more polluted study area). These differences could have been caused by men and women having different bone mineralization patterns, dietary habits, hormone concentrations, and occupational exposures. Unfortunately, these factors were not able to be analysed in this study.

We calculated of the Me_(tooth)_/Me_(bone)_ ratios for our samples. The ratios were higher for the samples from the residents of Bielsko-Biala than for the samples from the residents of Ruda Slaska, but the differences were not statistically significant (Table 9). However, the greatest differences between the ratios were obtained for lead (3,32 times higher ratio for the citizens of Bielsko-Biala), manganese (2,06 times higher ratio for the citizens of Bielsko-Biala) and copper (2,15 times higher ratio for the citizens of Bielsko-Biala). As these are the elements which easily substitute calcium in hydroxyapatite [19]. As bone reveals faster turnover of numerous elements, the lower levels of lead, manganese and copper are characteristic of the bones from the citizens of cleaner areas and influence significantly the Me_(tooth)_/Me_(bone)_ ratios. It would be valuable to compare the ratios obtained from our study to the results from other researchers, but to our best knowledge this is the first research work analysing occurrence of heavy metals in impacted wisdom teeth and the bone surrounding the teeth derived from the same individuals. The correlation analysis was performed to allow relationships between the metal concentrations in the samples to be assessed and interrelationships to be identified (Table 10). A significant positive correlation was only found between the cadmium concentrations in the tooth and bone samples from the inhabitants of Ruda Slaska (the Pearson’s correlation coefficient was 0.36). This indicated that the cadmium concentration in the tooth structure increased as the cadmium concentration in the surrounding bone increased. There could be closer relationships between heavy metal concentrations in teeth and mandibular bones in areas that are heavily polluted with heavy metals, although discrimination mechanisms tend to break down when an organism is exposed to high concentrations of heavy metals, and this decreases the number of correlations that are observed in tissues within the organism [19].

### Strengths and limitations

The original aspect of the study was the analysis of heavy metals in impacted wisdom teeth and the mandibular bones surrounding the impacted teeth. To the best of our knowledge, no previous studies of heavy metals in these tissues in relation to the concentrations in the environment have been performed. However, there are numerous research works on occurrence of heavy metals in tooth hard tissues only, but they do not contain any information about metal concentration in bone surrounding the teeth [38–40]. Only a small number of samples could be analysed because of the generally limited indications for the surgical removal of wisdom teeth. We only analysed samples from people who declared that they had never worked in industries involving increased exposure to heavy metals, had never smoked cigarettes, did not regularly take medication or dietary supplements, and ate only food from shops (i.e., not grown on their own land). The participants all declared that they had not been regularly exposed to second-hand cigarette smoke recently, but most could not be sure that they had not been regularly exposed to second-hand smoke in childhood. These data were collected from the participants’ medical histories, and no additional medical examinations were performed. These are undoubtedly important limitations of the study. It seems that impacted teeth and the surrounding alveolar bone can be used to assess environmental exposure to heavy metals in an area in which a person lives. This conclusion, however, must be verified in future research projects designed to exclude the possibility of additional dietary, occupational, and other types of exposure to heavy metals.

## Conclusions

The results of this study indicated that impacted mandibular teeth and the surrounding mandibular bone may reflect the environmental exposure of a person to cadmium and lead. An impacted tooth is isolated from the oral environment, so the elemental composition of the tooth will represent only elements delivered in blood while the tooth was forming. It is possible to obtain bone from around an impacted tooth when the tooth is surgically removed. The physiological and histological characteristics of bones and teeth mean that elements will be turned over more quickly in bone than in teeth. In this study, we found significant differences between the cadmium, chromium, copper, lead, manganese, and zinc concentrations in mandibular bones from people living in two areas in southern Poland with different environmental burdens of the heavy metals. The metal concentrations in the impacted teeth and surrounding bones did not correlate except for the cadmium concentrations in the samples provided by people living in the more polluted area (Ruda Slaska). More research is required to prove that impacted mandibular teeth and the surrounding bones can be used to assess environmental exposure to heavy metals. Future studies must exclude sources other than the blood of heavy metals to impacted teeth and the surrounding bones.

## Abbreviations

Me_(bone)_: Metal concentration in bone surrounding the impacted tooth
Me_(tooth)_: Metal concentration in an impacted tooth
PM10: Particulate matter smaller than 10 μm in diameter
